# MCT1 Is a New Prognostic Biomarker and Its Therapeutic Inhibition Boosts Response to Temozolomide in Human Glioblastoma

**DOI:** 10.3390/cancers13143468

**Published:** 2021-07-11

**Authors:** Vera Miranda-Gonçalves, Céline S. Gonçalves, Sara Granja, Joana Vieira de Castro, Rui M. Reis, Bruno M. Costa, Fátima Baltazar

**Affiliations:** 1 Life and Health Sciences Research Institute (ICVS), School of Medicine, University of Minho, Campus de Gualtar, 4710-057 Braga, Portugal; vera.miranda.goncalves@ipoporto.min-saude.pt (V.M.-G.); celinegoncalves@med.uminho.pt (C.S.G.); saragranja@med.uminho.pt (S.G.); joana.castro@i3bs.uminho.pt (J.V.d.C.); ruireis.hcb@gmail.com (R.M.R.); bfmcosta@med.uminho.pt (B.M.C.); 2 ICVS/3Bs-PT Government Associate Laboratory, 4805-017 Guimarães, Portugal; 3 Research Centre in Health and Environment (CISA), School of Health (ESS), Polytechnic Institute of Porto (P.PORTO), 4200-072 Porto, Portugal; 4 Department of Pathological, Cytological and Thanatological Anatomy, School of Health (ESS), Polytechnic Institute of Porto (P.PORTO), 4200-072 Porto, Portugal; 5 Molecular Oncology Research Center, Barretos Cancer Hospital, Barretos 14784-400, SP, Brazil

**Keywords:** monocarboxylate transporters, glioblastoma, lactate, Warburg effect, prognostic biomarker

## Abstract

**Simple Summary:**

Glioblastoma, the brain tumour with highest prevalence and lethality, exhibits a characteristic glycolytic phenotype with increased lactate production. Recently, we reported a MCT1 overexpression in GBMs tumours, being associated to tumour growth and aggressiveness. Thus, we aimed to disclose the role of MCT1 in GBM prognosis and in vivo therapy response. Importantly, MCT1 overexpression is associated with poor prognosis of GBM. Moreover, MCT1 inhibition retards GBM tumour growth and boosts response to temozolomide treatment.

**Abstract:**

*Background:* Glioblastomas (GBMs) present remarkable metabolism reprograming, in which many cells display the “Warburg effect”, with the production of high levels of lactate that are extruded to the tumour microenvironment by monocarboxylate transporters (MCTs). We described previously that MCT1 is up-regulated in human GBM samples, and MCT1 inhibition decreases glioma cell viability and aggressiveness. In the present study, we aimed to unveil the role of MCT1 in GBM prognosis and to explore it as a target for GBM therapy in vivo. *Methods:* MCT1 activity and protein expression were inhibited by AR-C155858 and CHC compounds or stable knockdown with shRNA, respectively, to assess in vitro and in vivo the effects of MCT1 inhibition and on response of GBM to temozolomide. Survival analyses on GBM patient cohorts were performed using Cox regression and Log-rank tests. *Results:* High levels of MCT1 expression were revealed to be a predictor of poor prognosis in multiple cohorts of GBM patients. Functionally, in U251 GBM cells, MCT1 stable knockdown decreased glucose consumption and lactate efflux, compromising the response to the MCT1 inhibitors CHC and AR-C155858. MCT1 knockdown significantly increased the survival of orthotopic GBM intracranial mice models when compared to their control counterparts. Furthermore, MCT1 downregulation increased the sensitivity to temozolomide in vitro and in vivo, resulting in significantly longer mice survival. *Conclusions:* This work provides first evidence for MCT1 as a new prognostic biomarker of GBM survival and further supports MCT1 targeting, alone or in combination with classical chemotherapy, for the treatment of GBM.

## 1. Introduction

Glioblastoma (GBM) is the most common and most lethal primary brain tumour in adults. Current treatment includes surgery, radiotherapy, and chemotherapy with the alkylating agent temozolomide (TMZ) [1], but despite treatment, the prognosis of patients is very dismal, with a median overall survival (OS) of approximately 15 months [2]. Critically, these figures have not changed significantly for decades, highlighting an urgent unmet need to develop novel therapeutic strategies for this cancer. To do so, it is crucial to identify novel clinically relevant biomarkers predictive of patient outcome and to explore how they can be therapeutically targeted.

Most cancer cells present a dynamic metabolic reprogramming, particularly on cellular energetics, displaying high glycolytic rates coupled with lactate production, even in the presence of normal levels of oxygen (Warburg effect). This has been recognized as an important hallmark of cancer [3], and confers advantages to cancer cells, including growth, survival and aggressiveness [4,5,6]. Concordantly, GBMs present increased glucose uptake compared to a normal brain [7], and ~90% of all glucose consumed by GBM cells is converted into lactate or alanine, which contributes to the remarkable infiltrative phenotype of GBM cells into the surrounding non-neoplastic tissue [8]. In this context, monocarboxylate transporters (MCTs) play an important role in the maintenance of high cancer cell glycolytic rates, contributing to acidification of the extracellular tumour microenvironment due to the proton-coupled mechanism of lactate transport [9]. The MCT family comprises 14 members with similar topology, but only the first four isoforms (MCT1-4) use lactate as common substrate [10,11,12,13,14,15]. Besides lactate, these MCT isoforms also mediate the transmembrane transport of other monocarboxylic acids, including pyruvate and ketone bodies (acetoacetate and d-β-hydroxybutyrate) [15]. MCTs have crucial roles in mammalian cell metabolism, are critical for metabolic communication between cells [11,16], and therefore present different kinetic characteristics and tissue distribution [10,13,17]. MCT1, MCT2, and MCT4 play a crucial role in the brain energetics, in the so called “astrocyte-neuron lactate shuttle”. Lactate produced by astrocytes leaves the cell through the activity of both MCT1 and MCT4, which is then utilized by neurons whose uptake is mediated by MCT2 [18]. The activity of these transporters in the brain has been demonstrated to play a role in learning and memory [19].

Upregulation of MCTs, especially MCT1 and MCT4, has been increasingly reported in different human solid tumours, demonstrating the importance of MCTs in cancer biology [20]. MCT1 and MCT4 overexpression has also been described in gliomas, being MCT1 isoform the most prevalent plasma membrane transporter responsible for lactate efflux [21,22]. We previously described the role of MCT1 in glioma cell survival and aggressiveness using in vitro and ex vivo models [21,23]. Other studies reported the potential of MCT1 targeting in reversing the cell growth and aggressiveness of several other malignant tumours, namely in diffuse large B cell lymphoma [24], oesophageal squamous cell carcinoma [25], breast [26], lung [27], and bladder cancer [28]. Thus, while MCTs are generally viewed as promising anticancer targets, additional studies are needed to clearly establish the prognostic value of MCTs in patients, and to support the therapeutic value of lactate transport inhibition in GBM. In the present study, we show that high MCT1 expression is a predictor of poor prognosis in multiple independent cohorts of GBM patients, establishing it as a novel biomarker. In addition, MCT1 knockdown reduces lactate efflux and cell aggressiveness in vitro and, more importantly, significantly increased the survival of orthotopic glioma in in vivo mice models. Critically, and from a translational perspective, MCT1 knockdown significantly increased the sensitivity of GBM cells to TMZ in vitro and in vivo, clearly extending the survival of mice.

## 2. Materials and Methods

### 2.1. Cells and Culture Conditions

U251 cells were kindly provided by Professor Joseph Costello, University of California, San Francisco, CA, USA. Cell line authentication was performed by IdentiCell Laboratories (Department of Molecular Medicine (MOMA) at Aarhus University Hospital Skejby in Århus, Denmark). Genotyping confirmed the complete identity of the cell line. Cells were maintained in Dulbecco’s Modified Eagle’s Medium (DMEM 1×, High Glucose; Gibco, Invitrogen, USA) supplemented with 10% Foetal Bovine Serum (FBS; Gibco, Invitrogen) and 1% penicillin/streptomycin solution (Gibco, Invitrogen, Waltham, MA, USA), at 37 °C and 5% CO_2_.

### 2.2. Generation of Stable shMCT1 Expressing Cells

For the generation of U251 cells stably expressing shMCT1, a pool of target-specific lentiviral vector plasmids each encoding 19–25 nt (plus hairpin) shRNAs to MCT1 knockdown (sc-37235-SH, Santa Cruz Biotechnology, Santa Cruz, CA, USA) were used. Transfection was done by using the FUGENE HD reagent (Roche, Switzerland), as recommended by the manufacturer. Cells were plated on 12 well plates until 80% of confluence and transfected in DMEM medium with FBS without antibiotic addition, for 24 h. After that, stable transfectants were selected with 1µg/mL puromycin for one month. The empty vector was also transfected as a control. For clone isolation, 200 U251 shMCT1 and U251 shCTRL cells were plated in a 100 mm plate and several clones were collected, expanded, and analysed for MCT1 expression by Western blot.

### 2.3. Drugs

Alpha-cyano-4-hydroxycinnamate (CHC; Sigma-Aldrich, St. Louis, MO, USA), Temozolomide (TMZ, Sigma-Aldrich), and AR-C155858 compound (R&D systems, Minneapolis, MN, USA) were dissolved in dimethyl sulfoxide (DMSO; Sigma-Aldrich, St. Louis, MO, USA) to 3 M, 100 mM, and 3 mM stock solutions, respectively.

### 2.4. Antibodies

The following antibodies and conditions were used for immunofluorescence (IF) and Western blot (WB) assays: MCT1 ((AB3538P, Chemicon International, MERCK, Germany (IF); 1:200), (H-1, sc-365501, Santa Cruz Biotechnology, USA (WB); 1:500)); MCT4 (H-90, sc-50329, Santa Cruz Biotechnology, USA; 1:500); hypoxia-inducible factor 1α (HIF-1α) (610958, BD Biosciences, Germany; 1:100 dilution (IF); 1:500 dilution (WB)), carbonic anhydrase IX (CAIX) (Abcam, UK, 1:2000), and hexokinase II (HKII) (Abcam, UK, 1:750).

### 2.5. GBM Patient Cohorts and Survival Analysis

The Cancer Genome Atlas (TCGA) data [29] was accessed through the GDC portal (https://portal.gdc.cancer.gov/), as explained in [30], to obtain MCT1 microarray expression data from GBM (*n* = 572) and LGG (lower grade glioma, WHO grades II and III) patients (*n* = 27), and non-cancer unmatched samples (*n* = 10). GBM patient clinical data was also collected. MCT1 expression and clinical data from Rembrandt (*n* = 203) [31], Ducray (*n* = 52) [32], Lee Y (*n* = 191) [33], Murat (*n* = 80) [34], Gravendeel (*n* = 159) [35], Joo (*n* = 54) [36], and Nutt (*n* = 28) [37] patient GBM datasets were also obtained, as previously described [30]. The maximally selected rank statistics [38] were used to determine an optimal cut-off for the survival analysis, as provided in the ‘survminer’ package.

### 2.6. Western Blot

Western blot was performed as described previously [19]. Primary antibodies were incubated overnight at 4 °C and bound antibodies were detected by chemiluminescence (Supersignal West Femto kit; Pierce, Thermo Scientific, Waltham, MA, USA) (Figure S1). β-Actin or tubulin were used as loading controls.

### 2.7. Immunofluorescence

Cells were grown on glass coverslips at a density of 2 × 10^4^ cells/well and incubated at 37 °C and 5% CO_2_ overnight. Then, cells were incubated in DMEM without FBS for 24 h. Immunofluorescence was performed as previously described [26]. Briefly, slides were incubated with the primary antibodies (room temperature, overnight), and then incubated with the secondary antibody anti-rabbit-Alexa Fluor 488 (A11008, Invitrogen, Waltham, MA, USA, 1:500) for 1 h in 5% BSA (MCT4 and MCT1), or the secondary antibody anti-rabbit-Alexa Fluor 594 (A11032, Invitrogen USA, 1:250) (HKII and HIF-1α). Images were acquired by a fluorescence microscope (Olympus IX81) with the Cell P software.

### 2.8. Cell Metabolism Assays

Cells were plated in 48 well plates at a density of 3 × 10^4^ cell per well. Then, they were cultured in DMEM at 4.5 g/L glucose without FBS, untreated or in the presence of 10 mM CHC. Glucose and lactate contents in the cell culture media were quantified after 24 h and 48 h, with the commercial kits Spinreact, Spain and Roche, Switzerland respectively), as described in [21]. Results are shown as total µg/total biomass, assessed by the sulforhodamine B assay (SRB, TOX-6, Sigma-Aldrich, USA).

### 2.9. Cell Viability Assay

To determine the response of U251 shMCT1 knockdown to CHC, AR-C155858, and TMZ, cell viability was estimated using the Sulphorhodamine B assay, following the manufacturer’s instructions, as described in [26]. U251 shMCT1 and U251 shCTRL cells were plated into 96-well plates, at a density of 3 × 103 cells/well, in DMEM medium, and treated with different concentrations of CHC or AR-C155858 for 24 h, 48 h, and 72 h. Additionally, TMZ treatment was performed for 72 h, as well as combinatory AR-C155858 + TMZ treatment. Spectrophotometric measurements were done at 490 nm, using 655 nm as reference absorbance (Tecan infiniteM200). Results represent the mean of three independent experiments, each one in triplicate, and were analysed using the Graph Pad Software.

### 2.10. In Vivo Orthotopic GBM Xenografts

All animal experiments (immunocompromised NSG mice, NOD.Cg-Prkdcscid Il2rgtm1Wjl/SzJ) were approved by the national ethical committee (Direção Geral de Alimentação e Veterinária, Portugal) and were performed in accordance with the European Union Directive 2010/63/EU. For intracranial models, a total of 5 × 10^5^ U251 cells, namely U251 shCTRL (*n* = 11) and U251 shMCT1 (*n* = 11), were injected in the brain striatum (1.8 mm medial-lateral right, 0.1 mm anterior-posterior, and 2.5 mm dorsal-ventral from the bregma) of 2–6 months aged mice as previously described [30]. TMZ treatment of U251 shCTRL (*n* = 5) and U251 shMCT1 (*n* = 5) mice started at day 15 after U251 cells implantation. Each mouse was treated daily with 50 mg/kg TMZ by oral gavage, in 2 cycles (5 days ON, 3 days OFF) [39]. Animal body weight was assessed 3 times a week, and general behaviour and symptomatology was evaluated daily. Mice were sacrificed when body weight reached ≤70% of their maximum body weight, perfused with saline solution followed by PFA 4%, and whole brains were collected and paraffin embedded for subsequent immunohistochemistry analyses.

### 2.11. Immunohistochemistry

MCT1, MCT4, CAIX and Ki67 protein expression for U251 shMCT1 and U251 shCTRL tumours collected from NSG mice were evaluated by immunohistochemistry (IHC). IHC for MCT1 was performed using UltraVision LP detection system HRP Polymer (Thermo Fisher Scientific, Waltham, MA, USA), and for MCT4, CAIX and Ki67 using UltraVision Large Volume Detection System Anti-Polyvalent, HRP (Thermo Fisher Scientific), as previously described [21,23]. Briefly, deparaffinised and rehydrated slides were submitted to heat-induced antigen retrieval for 20 min at 98 °C with 10 mM citrate buffer (pH 6.0). After endogenous peroxidase inactivation, incubation with the primary antibody was performed overnight for MCT1, and during 2 h for MCT4, CAIX and Ki67, at room temperature. The immune reactions were visualized with 3,3′-diamonobenzidine (DAB + Substrate System; Dako, Denmark) as a chromogen. The slides were counterstained with haematoxylin and mounted with Entellan^®^ (Merck-Millipore, Darmstadt, Germany). For each immunoreaction, a positive control was included.

### 2.12. Statistical Analysis

The GraphPad prism 5 software was used for statistical analysis, with the Student *t* test used for in vitro studies and log-rank test for mice survival analysis, considering significant values *p* < 0.05. SPSS 22.0 software (SPSS, Inc., Chicago, IL, USA) was used to evaluate the prognostic value of MCT1 in GBM patients by the log-rank test. Cox proportional hazard model was used to perform multivariate analysis (in SPSS 25 software; SPSS, Inc., Chicago, IL, USA), where the potential confounding effects of age and gender were considered. Comprehensive Meta Analysis (CMA) software (Biostat, Inc., Englewood, NJ, USA) was used to perform meta-analysis. For this, hazard ratios and 95% confidence intervals were used. A random effects statistical model was applied.

## 3. Results

### 3.1. Increased MCT1 Expression Is a Predictor of Poor Prognosis in GBM Patients

**Figure 1:**
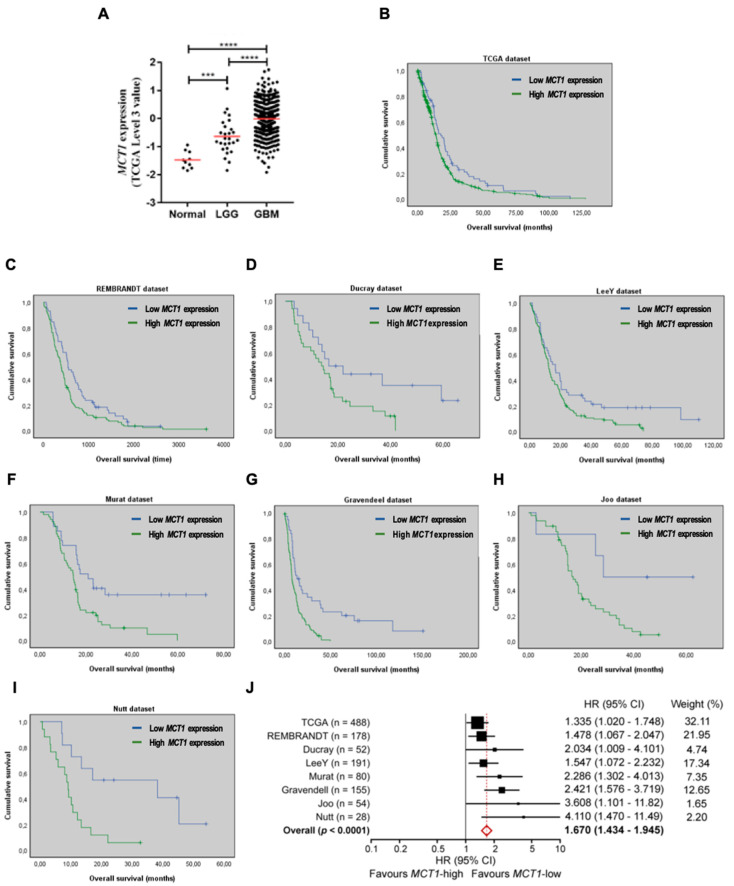
*MCT1* is over-expressed in GBM patients and associates with poor prognosis. (**A**) *MCT1* expression level in 10 unmatched normal brains, 27 lower-grade gliomas (LGG), and 572 glioblastomas (GBM) patients from TCGA; *** *p* < 0.001 and **** *p* < 0.0001. (**B**–**I**) Kaplan-Meier survival curves of *MCT1*-low and *MCT1*-high GBM patients derived from microarray data from (**B**) TCGA dataset, *n* = 488, median OS 17.6 vs. 13.9 months, low vs. high *MCT1* expression, respectively; *p* = 0.034; (C) REMBRANDT dataset, *n* = 178, median OS 541 vs. 390 days (*p* = 0.018); (**D**) Ducray dataset, *n* = 52, median OS 16.5 vs. 13.7 months (*p* = 0.043); (**E**) Lee Y dataset, *n* = 191, median OS 16.6 vs. 12.2 months (*p* = 0.018); (**F**) Murat dataset, *n* = 80, median OS 20.9 vs. 14.4 months (*p* = 0.003); (**G**) Gravendeel dataset, *n* = 155, median OS 13.3 vs. 7.8 months (*p* = 0.000031); (**H**) Joo dataset, *n* = 54, median OS 28.4 vs. 16.9 months (*p* = 0.022); (**I**) Nutt dataset, *n* = 28, median OS 38.2 vs. 9.1 months (*p* = 0.004); and (**J**) Forest plot of hazard ratios (HR), demonstrating the relationship between *MCT1* expression and GBM patients’ overall survival. The HR for each cohort is represented by a black square (its size represents the weight of the study for the meta-analysis) and 95% confidence intervals (CI) by the extending lines. The estimated pooled effect is represented by a red diamond (HR = 1.670; 95% CI, 1.434–1.945; *p* < 0.0001).

A previous study from our group showed that MCT1 expression is associated with growth and aggressiveness of GBM models [21], but its relevance as a potential prognostic biomarker in patients remains unknown. Therefore, we started by investigating if MCT1 expression can be associated with the malignancy grade of gliomas and whether it is predictive of the overall survival (OS) of GBM patients using a variety of independent cohorts. We found that MCT1 expression increases significantly from normal brain samples to LGG (lower grade glioma), and to GBM patients from TCGA (Figure 1A). Critically, high *MCT1* expression was significantly associated with a shorter OS of GBM patients in the large TCGA cohort (Figure 1B). This prognostic value of *MCT1* in GBM patients was then consistently validated in seven additional cohorts of patients (Figure 1C–I). This finding was then validated in the TCGA dataset (where other clinical information, such as age and gender are available), using a multivariable Cox model, showing that *MCT1*-high expression is associated with a shorter OS, independently of other prognostic variables, including patient age and gender (Table 1). A meta-analysis including these eight datasets showed that overall *MCT1*-high expression is associated with the shorter survival of GBM patients (Figure 1J). Together, these data clearly establish MCT1 as a novel prognostic biomarker with clinical relevance for GBM patients.

### 3.2. MCT1 Downregulation Alters GBM Cell Energetic Metabolism and Growth

**Figure 2:**
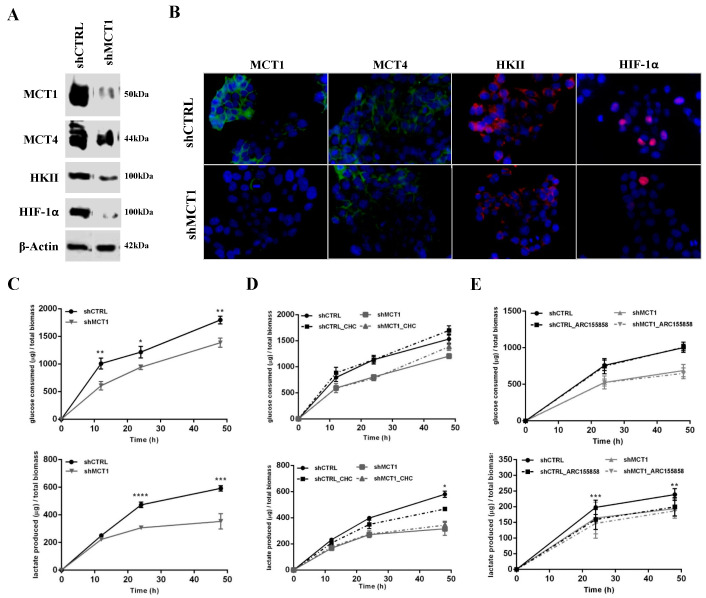
Effect of MCT1 downregulation on cell metabolism. Expression of MCT1, MCT4, HKII and HIF-1α in U251 shMCT1 cells by (**A**) Western Blot and (**B**) Immunofluorescence; Western blot MW: HIF-1α 100 kDa, HKII 100 kDa, MCT1 50 kDa, MCT4 44 kDa and β-Actin 42 kDa; Representative pictures was taken at 400× magnification. (**C**) Glucose consumption and lactate production in MCT1 knockdown cells, up to 48 h; * *p* < 0.05; ** *p* < 0.01; *** *p* < 0.001; **** *p* < 0.0001 U251 shCTRL vs. U251 shMCT1. (**D**) Glucose consumption and lactate production in MCT1 knockdown cells treated with CHC, up to 48 h; * *p* < 0.05 U251 shCTRL vs. U251 shCTRL + CHC (**E**) Glucose consumption and lactate production in MCT1 knockdown cells treated with ARC155858, up to 48 h; ** *p* < 0.01; *** *p* < 0.001 U251 shCTRL vs. U251 shCTRL + ARC155858. Results are representative of three independent experiments, each in triplicate.

In order to explore MCT1 as a therapeutic target in GBMs, we silenced MCT1 in U251 cells. We confirmed U251 shMCT1 cells present very low levels of MCT1 expression, as compared to shCTRL cells (Figure 2A). Importantly, this was not accompanied by any compensatory increase in MCT4 expression (Figure 2A). Additionally, to evaluate the effect of MCT1 downregulation on glycolytic and hypoxic phenotypes, HKII and HIF-1α expression was assessed. U251 shMCT1 cells display a decreased expression of HKII and HIF-1α proteins (Figure 2A), suggesting a reprogramming of the glycolytic metabolic profile. Further, we also assessed their expression and cellular localization in U251 shMCT1 by immunofluorescence (IF). Very low MCT1 expression in U251 shMCT1 was also confirmed by IF, while the levels of MCT4 expression in the plasma membrane were maintained regardless of MCT1 silencing (Figure 2B). The expression of nuclear HIF-1α and cytoplasmic HKII also decreased in U251 shMCT1 cells (Figure 2B).

In what concerns the metabolic behaviour, there was a decrease in glucose uptake and lactate release in U251 shMCT1 at 24 h and 48 h, as compared to control cells (U251 shCTRL; Figure 2C). Of note, MCT1 silencing rendered cells insensitive to the MCT1 inhibitors CHC (Figure 2D) and AR-C155858 (Figure 2E), since no alterations in lactate release were observed, as opposed to U251 shCTRL cells (Figure 2D,E).

**Figure 3:**
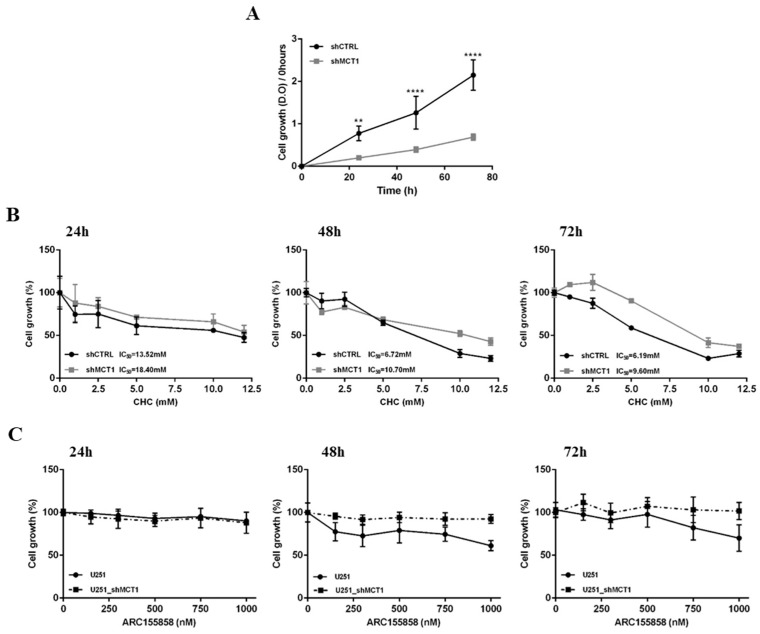
MCT1 downregulation in glioma cell growth, CHC and AR-C155858 response. (**A**) Cell growth of U251 shCTRL and U251 shMCT1 over time. (**B**) Response of U251 shMCT1 cells to CHC by cell viability assay; IC50 values mean are representative of three independent experiments. (**C**) Response of U251 shMCT1 cells to ARC155858 by cell viability assay. Results are representative of three independent experiments, each in triplicate; ** *p* < 0.01; **** *p* < 0.0001 U251 shCTRL vs. U251 shMCT1.

Furthermore, MCT1 downregulation decreased significantly cell growth over time (Figure 3A), and decreased the sensitivity to CHC (as expressed by higher IC50 values), compared to control cells (Figure 3B). Additionally, MCT1 downregulation rendered U251 shMCT1 cells insensitive to AR-C155858 at 48 h and 72 h (Figure 3C), validating MCT1 as target. Globally, our data suggests that MCT1 silencing in GBM reduces the expression of molecular mediators of glycolytic metabolism, resulting in impaired energetic metabolism and insensitivity to pharmacological inhibitors of MCT1.

### 3.3. MCT1 Downregulation Increases Sensitivity to TMZ In Vitro and In Vivo, Increasing GBM Mice Model Survival

**Figure 4:**
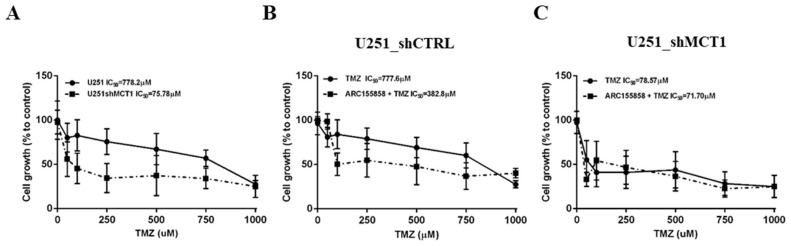
MCT1 inhibition effect on glioma cells TMZ response. (**A**) Response to TMZ in U251 shCTRL and U251 shMCT1; Effect of 300 nM AR-C155858 in TMZ response for U251 shCTRL (**B**) and U251 shMCT1 (**C**). IC50 values mean are representative of three independent experiments.

We next investigated the role of MCT1 in the sensitivity of GBM cells to the standard-of-care chemotherapeutic drug TMZ, using in vitro and in vivo experiments. Firstly, the genetic downregulation of MCT1 in U251 shMCT1 cells significantly increased the sensitivity to TMZ, strikingly decreasing TMZ IC50 value from 778.2 µM to 75.78 µM (*p* = 0.0159; Figure 4A). To further support these findings, a pharmacological approach targeting MCT1 was then tested in combination with TMZ treatment. Consistently, U251 shCTRL cells treated with MCT1 inhibitor AR-C155858 were significantly more sensitive to TMZ treatment, while this enhanced effect was not observed in U251 shMCT1 cells (AR-C155858 + TMZ IC50 values of 382.8 µM and 71.7 µM, respectively; Figure 4B). Additionally, no effect was observed on TMZ response when U251 shMCT1 cells were treated with the MCT1 specific inhibitor (300 nM AR-C155858) compared with TMZ alone (Figure 4C). An important gene that is associated with TMZ sensitivity in GBM is the DNA-repair gene O6-methylguanine–DNA methyltransferase (MGMT). Thus, in an attempt to explain the increased sensitivity to TMZ upon MCT1 silencing, we investigated the expression of MGMT in U251 cells. Importantly, U251 shMCT1 cells exhibited a decrease in MGMT transcriptional levels (Figure S2A), but with no significant alterations at the MGMT protein level (Figure S2B) when compared to U251 shCTRL cells. 

**Figure 5:**
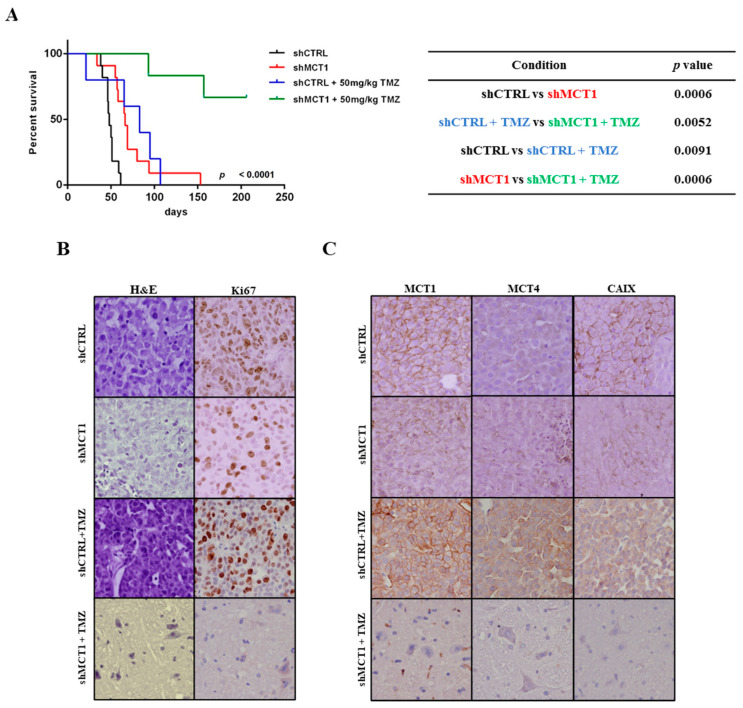
In vivo effect of MCT1 knockdown in GBMs overall survival. (**A**) Kaplan-Meier survival curves for in vivo orthotopic intracranial GBM model. Log-rank test shMCT1 vs. shCTRL vs. shMCT1+ TMZ vs. shCTRL+ TMZ, *p* < 0.0001 *n* = 11; *n* = 11; *n* = 5; *n* = 5 per group, respectively; (**B**) Hematoxylin-eosin staining of NSG mice brains; Representative pictures of immunohistochemistry for Ki67 in brain tissues of NSG mice at 400× magnification. (**C**) MCT1, MCT4 and CAIX expression in shCTRL and shMCT1 plus TMZ in brain tissues at 400× magnification.

In order to confirm the potential prognostic value of MCT1 observed in GBM patients, we established an intracranial orthotopic GBM xenograft model with U251 shCTRL and U251 shMCT1 cells in NSG mice. Consistent with the patient data, we observed that animals bearing U251 shMCT1 tumours present a significant increase in OS compared to U251 shCTRL tumours (median OS of 63.5 days and 43.5 days, respectively: Figure 5A). Histopathological and IHC analysis for Ki67 showed a lower proliferative index for shMCT1 compared to shCTRL (Figure 5B). Long-term MCT1 knockdown in vivo was also confirmed by IHC in the NSG mice U251 shMCT1 cell tumours (Figure 5C), while MCT4 expression was similar between U251 shCTRL vs. shMCT1 (Figure 5C), consistent with the in vitro findings (Figure 2A,B). Additionally, the effect of MCT1 downregulation on tumour hypoxia markers was characterized by an assessment of expression of the HIF-1α downstream target CAIX. In Figure 5C, it can be seen that CAIX expression decreased for NSG mice U251 shMCT1 tumours compared to U251 shCTRL condition.

Having established the impact of MCT1 downregulation in treatment-naïve conditions, we then tested the impact of MCT1 in the response of GBM to TMZ (orthotopic intracranial GBM model). We found that animals bearing U251 shMCT1 tumours treated with TMZ presented a prominent and statistically significant increased overall survival (OS) as compared to U251 shCTRL TMZ-treated mice (Figure 5A). In addition, at the experimental endpoint (206 days), all U251 shCTRL mice were dead, while four out of six mice of U251 shMCT1 group were still alive and without any GBM-related weight loss or neurological symptoms. Histopathological analysis revealed the absence of tumoural masses in U251 shMCT1 group treated with TMZ (Figure 5B). The absence of Ki67 staining observed in animals bearing U251 shMCT1 cells exposed to TMZ treatment support these findings (Figure 5B). The absence of MCT1 and CAIX expressions was confirmed in animals bearing U251 shMCT1 cells with TMZ treatment (Figure 5C).

## 4. Discussion

Glycolytic metabolism has recently been recognized as a fundamental mechanism in the metabolic reprogramming of cancer cells [3]. The large amounts of glucose consumed by tumour cells have been useful in the diagnosis of cancer using ^18^F-fluoro-2-deoxy-d-glucose positron emission tomography (_18_FDG PET) scanning, particularly in the detection of metastases and recurrent disease, and to monitor therapy response [40]. To sustain the glycolytic phenotype of tumour cells, several proteins are differentially expressed, like proteins of the glycolysis pathway and some pH regulators, including MCTs [17,23].

GBM is the most prevalent and also the most aggressive malignant primary brain tumour of the central nervous system in adults [41]. These tumours present metabolic reprogramming, with high glycolytic activity and consequent increased lactate production [9]. Our group has previously shown MCT1 and MCT4 upregulation in a human series of GBM samples, being MCT1 the most prevalent plasma membrane transporter [21]. Additionally, we demonstrated that MCT1 downregulation or activity inhibition disturbs the hyper-glycolytic phenotype of GBM cell lines [21]. Moreover, MCT1 activity has been associated with angiogenesis [42,43], and with bevacizumab therapy response in vitro [44]. Importantly, we showed that tumour hypoxia leads to the up-regulation of MCT1, but not of MCT4, in a series of GBM patients [23]. MCT4 has been recognized as the major mediator of lactate efflux to the tumour microenvironment under hypoxic conditions, and as a malignant contributor in several tumours. However, our previous studies reinforce the importance of MCT1 as the major gatekeeper of lactate efflux in GBM, being a promising candidate for anti-GBM therapy. In the present study, we intended to evaluate, beyond tumour growth and aggressiveness in GBM, the prognostic value of MCT1 and its role in the sensitivity to TMZ response in patients and also in an orthotopic xenograft GBM model.

Downregulation of MCT1 led to a decrease in glucose consumption and lactate production, in accordance with what was previously reported in a breast cancer model [26,45], as well as in colorectal [46] and bladder cancer [24,28] among others. Additionally, downregulation of MCT1 in U251 cells compromises the response to CHC and to the MCT1 specific inhibitor AR-C155858, supporting MCT1 as target of these drugs. These data support the important role of MCT1 in the maintenance of the glycolytic phenotype of GBM cell lines, as opposed to other studies that identify MCT4 as the most important isoform involved in the maintenance of the glycolytic activity of tumour cells [47].

Notwithstanding, to the best of our knowledge, the prognostic value of MCT1 in GBM has not been previously reported. Testing a large variety of independent cohorts of GBM patients, we found high MCT1 expression is associated with a shorter OS of GBM patients. Concordantly, we found that MCT1 downregulation in GBM is sufficient to significantly increase the survival of mice bearing brain tumours. Together, our clinical, in vitro, and in vivo results emphasize the role of MCT1 as a valid prognostic biomarker for GBM. Although not yet described for GBM, few studies have explored the prognostic value of MCT1 in other tumour types. For example, a study in bladder cancer showed that high MCT1 expression associates with shorter OS compared with the low MCT1 expression group [28]. Similar associations were reported in patients with endometrial cancer [48] and clear cell renal carcinoma [49]. Beyond those described by our group in GBM [21], few other studies have reported that inhibition of MCT1 decreases GBM cell proliferation and invasion, promotes cell death [50,51], and increases the sensitivity to radiotherapy [52].

Further, the lower U251 shMCT1 proliferative capacity suggests that MCT1 may have an important role in tumour growth capacity. Several studies reported the importance of glioma stem cells on tumour initiation [53]. However, at the moment, few studies have described the metabolic profile of glioma stem cells (GSC). A study in a murine glioblastoma model using neural stem cells showed that these cells have the capacity of tumour formation, and present a metabolic signature associated with a glycolytic phenotype with increased expression of LDHA [54]. Additionally, it was verified that these stem cells present higher extracellular acidification than control conditions [54]. In fact, Takada et al. showed that MCT1 is the most prevalent MCT isoform in GSC [55]. Additionally, MCT inhibition decreases GSC proliferation, as well as sphere formation capacity in U251 cells [55]. Thus, an association of MCT1 in glioma initiation capacity could emerge as a new hypothesis. To consolidate that, the role of MCT1 in in vitro glioma sphere formation capacity and limiting dilution assay should be explored in the near future to support this hypothesis.

Despite the availability of new molecular targeted therapies, the standard GBM therapy remains maximal safe surgical resection, followed by radiotherapy and concomitant chemotherapy (TMZ) [56]. The present study showed the involvement of MCT1 in the sensitivity of GBM to TMZ, as MCT1 downregulation promoted an increased TMZ response both in in vitro and in vivo models. To support the clinical translation of these findings, in vivo combination of MCT1 targeting drugs in GBM models should be pursued, alone or in combination with TMZ. The MCT1 inhibitor AZD3965 has been recently taken to Phase I/II clinical trials, for patients with advanced cancers (clinicaltrials.gov NCT01791595). Despite the ubiquitous MCT1 expression in several human tissues, during the trials, the drug demonstrated to be well tolerated, with nausea and fatigue being the most commonly observed side effects [57]. An expected on-target effect was also reported in retinal function, but changes were reversible. However, knowledge on long term effects of MCT1 inhibition needs further investigation.

Since the activity of the DNA-repair protein MGMT is negatively associated with TMZ sensitivity in GBM, we investigated the expression of MGMT in U251 cells upon MCT1 silencing. Despite the decrease in MGMT transcriptional levels (RNA), we saw no significant alterations at the protein level. Thus, this does not seem to be the mechanism by which MCT1 downregulation boosts the effect of TMZ. Another possible mechanism could be related with the role of MCTs in the acidification of the tumour microenvironment [58]. A decrease in TMZ IC50 for U251 shMCT1 cells, as well as an increased OS in animals injected with U251 shMCT1 followed TMZ treatment could be partly explained by a lower lactic acid efflux in U251 shMCT1 cells. TMZ is administered orally in patients, is stable at acidic pH (e.g., stomach), and is spontaneously converted, at neutral or slightly basic pH (pH~7.4) to its active metabolite 5-(3-methyltriazen-1-yl)imidazole-4-carboxamide (MTIC), which reacts with DNA to form O6-methylguanine adducts, promoting DNA damage and subsequent cell death [59]. Thus, the activation of TMZ may be compromised by the acidic microenvironment created by GBM cells, which stabilizes the molecule. Therefore, by compromising the activity of MCT1 (shMCT1), the extracellular pH of the cancer cell would be less acidic, and this could consequently promote the conversion of the TMZ prodrug to its active metabolite.

## 5. Conclusions

MCT1 is a novel biomarker of prognosis in GBM and at the same time is an attractive therapeutic target, whose downregulation prevents tumour growth, improves mice survival, and boosts the therapeutic response to TMZ therapy.

## Figures and Tables

**Table 1:** Cox multivariable survival analysis in GBM patients from TCGA.

Parameters	Overall Survival (OS)	Hazard Ratio	95% CI
	*p*-Value		
*MCT1* expression ^a^	**0.032**	1.345	1.025–1.764
Age at diagnosis ^b^	**<0.0001**	1.827	1.499–2.226
Gender ^c^	0.117	1.175	0.961–1.437

^a^ *MCT1*-low (*n* = 71) vs. *MCT1*-high (*n* = 417) expression. ^b^ Age below average (average = 58; *n* = 237) vs. Age above average (*n* = 251). ^c^ Female (*n* = 187) vs. Male (*n* = 301). Bold-faced values indicate significant *p*-values.

## Data Availability

Publicly available datasets were analyzed in this study. This data can be found at: https://portal.gdc.cancer.gov/ and http://gliovis.bioinfo.cnio.es/.

## Supplementary Materials

The following are available online at https://www.mdpi.com/article/10.3390/cancers13143468/s1, Figure S1: Original Western blot pictures; Figure S2: MGMT expression in U251 shMCT1 cells.

## Abbreviations

GBM (glioblastoma); TMZ (temozolomide); monocarboxylate transporter (MCT); CHC (alpha-cyano-4-hydroxycinnamate); OS (overall survival), HR (hazzard ratio).
