# Supplementary material for: MCT1 Is a New Prognostic Biomarker and Its Therapeutic Inhibition Boosts Response to Temozolomide in Human Glioblastoma
Source: Cancers (Basel). 2021 Jul 11;13(14):3468. doi: 10.3390/cancers13143468 (PMC8306807; doi:10.3390/cancers13143468)
Supplement: Supplementary file 1 [file cancers-13-03468-s001.zip › cancers-1245751-supplementary.pdf]

Supplementary Materials

MCT1 Is a New Prognostic Biomarker and Its Therapeutic Inhibition Boosts Response to Temozolomide in Human Glioblastoma

Vera Miranda-Gonçalves, Céline S. Gonçalves, Sara Granja, Joana Vieira de Castro, Rui M. Reis, Bruno M. Costa and Fátima Baltazar

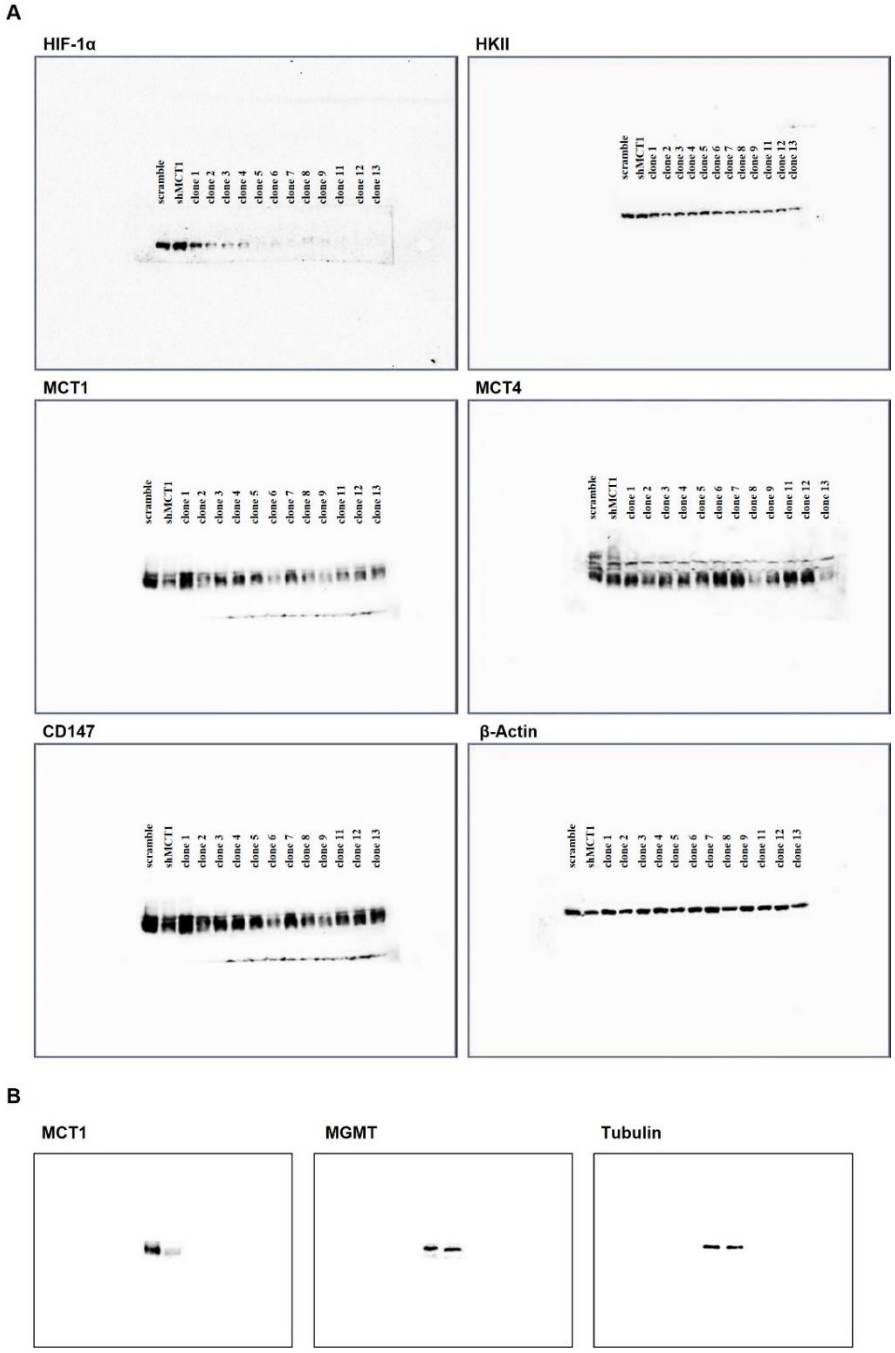

Figure S1. Original Western blot pictures**.** To MCT1, MCT4, CD147, HKII and HIF-1α proteins, the U251 scramble and U251 shMCT1 clone 9 were selected for final picture represented in Figure 2 of the manuscript **(A)**; MCT1 and MGMT expression in U251 scramble and U251 shMCT1 cells represented in supplementary Figure S1 **(B)**.

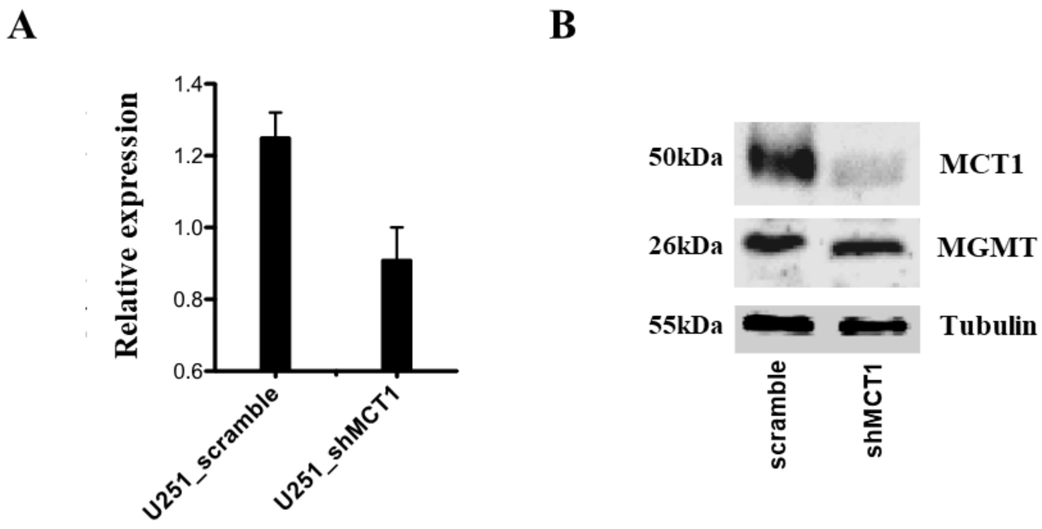

Figure S2. MGMT expression in U251 shMCT1 cells. Relative MGMT expression in U251 shMCT1 cells at transcriptional level by real time PCR **(A)** and at protein level by WB **(B)**. Results are representative of three independent experiments. WB molecular weights: MCT1, 50kDa and MGMT, 26kDa.
